# Pioneering robotic-assisted surgery for insulinoma during pregnancy: The first case report and literature review

**DOI:** 10.1016/j.heliyon.2024.e34239

**Published:** 2024-07-06

**Authors:** Voraboot Taweerutchana, Sawaraj Choksakunwong, Amornrat Lerwattrakarn, Wipapat Vicki Chalermwai, Thita Intralawan, Raweewan Lertwattanarak

**Affiliations:** a Minimally Invasive Surgery Unit, Division of General Surgery, Department of Surgery, Faculty of Medicine Siriraj Hospital, Mahidol University, Bangkok, Thailand; b Department of Pathology, Faculty of Medicine Siriraj Hospital, Mahidol University, Bangkok, Thailand; c Division of Endocrinology and Metabolism, Department of Medicine, Faculty of Medicine Siriraj Hospital, Mahidol University, Bangkok, Thailand; d Diabetes, Thyroid, and Endocrine Clinic, Siriraj Piyamaharajkarun Hospital, Faculty of Medicine Siriraj Hospital, Mahidol University, Bangkok, Thailand

**Keywords:** Insulinoma, Pregnancy, Robotic enucleation, Case report

## Abstract

**Introduction:**

Insulinoma during pregnancy is a rare condition with vague clinical symptoms, making diagnosis challenging. The standard treatment for insulinoma is surgical tumor removal, preferably using a minimally invasive method. However, there have been no recorded examples of employing a robotic platform in pregnant women with insulinoma. In this report, we present the first successful case of robotic enucleation for insulinoma during pregnancy.

**Case presentation:**

A 30-year-old pregnant woman presented with recurrent hypoglycemic symptoms throughout her first trimester that were relieved by food intake. After confirming endogenous hyperinsulinemia, an abdominal magnetic resonance imaging scan was performed to locate the tumor. A well-defined 2-cm mass was found in the pancreatic body. Robotic enucleation was performed at week 18 of gestation, and the patient experienced relief from hypoglycemic episodes postoperatively. Her blood glucose levels returned to normal, and she had an uneventful pregnancy. The patient eventually delivered a healthy baby via cesarean section without any complications.

**Conclusions:**

For a subset of pregnant individuals with insulinoma, a minimally invasive approach as robotic-assisted surgery is safe and feasible. This innovative technique has the potential to both mothers and fetuses.

## 1 introduction

Insulinomas are rare neuroendocrine tumors that predominantly occur in the pancreas and secrete insulin, leading to hypoglycemia [1]. They are the most common type of functional pancreatic neuroendocrine tumor. The typical symptoms included episodes of hypoglycemia, such as sweating, palpitation, tremors, confusion, and, in severe cases, loss of consciousness [2]. The annual incidence of insulinoma is reported to be between 0.67 and 4 cases per million individuals [3]. Insulinoma during pregnancy is an extremely uncommon occurrence. In some pregnant patients, the clinical symptoms of hypoglycemia can be nonspecific. Therefore, the diagnosis of hypoglycemia in these cases requires confirmation based on Whipple's triad. Whipple's triad includes the presence of hypoglycemic symptoms, a low plasma glucose concentration (less than 3 mmol/L in nondiabetic patients), and the resolution of hypoglycemic symptoms upon the restoration of plasma glucose concentration [2]. The biochemical diagnosis of insulinoma is typically established through the demonstration of hypoglycemia resulting from excessive endogenous insulin production [4].

Hypoglycemia during pregnancy can have detrimental effects on both maternal and fetal outcomes. It can lead to abnormal weight gain, cardiac arrhythmias, aberrant behavior, and reduced awareness of subsequent hypoglycemic episodes [5]. The association between severe hypoglycemic episodes and the development of congenital malformations in fetuses is not well understood [6]. Infants exposed to hypoglycemia during pregnancy have been found to have a smaller head circumference, lower birth weight, and shorter body length than those with normal glucose levels [7]. Insulinoma is a rare cause of hypoglycemia during pregnancy, with fewer than 40 cases reported in the literature [[8], [9], [10], [11], [12], [13], [14], [15], [16], [17], [18], [19], [20], [21], [22], [23], [24], [25], [26], [27], [28], [29], [30], [31], [32]]. The timing of diagnosis is crucial as it determines the optimal timing for surgery. Diagnosing insulinoma in early pregnancy can be challenging due to symptoms such as nausea, fatigue, and mild hypoglycemic episodes. Factors such as radiation exposure and the use of iodinated contrast media are also essential considerations for tumor localization. The timing of surgery depends on factors such as gestational age, severity of hypoglycemia, and the complexity of therapy.

Laparoscopic surgery is widely regarded as the gold standard in many fields of surgery, yet it has limitations. While it offers benefits such as less postoperative pain, shorter hospital stays, and quicker recovery compared to open surgery, it also minimizes tissue damage, which lowers the risk of infections [33]. However, it has some drawbacks, including longer operating times, no tactile sensation (lack of haptic feedback), and limited dexterity.

Robotic surgery enhances laparoscopic techniques by offering three-dimensional visualization, improved precision, and reduced tremors. It provides better ergonomics for surgeons, allowing for greater control and dexterity during the procedure. The robotic system is particularly effective in narrow spaces, which is advantageous when the surgical field is restricted. Despite these advantages, robotic surgery comes with higher costs [34,35].

This is the first case report of robotic-assisted pancreatic enucleation for insulinoma in a pregnant woman. The robotic approach was particularly beneficial in this case due to the patient's pregnancy, providing improved visibility and ergonomics essential for delicate dissection in the presence of an enlarged uterus. The ability of the robotic system to operate effectively in restricted spaces was crucial, as the enlarged uterus limited the available surgical space. Reports indicate that robotic surgery during pregnancy is both safe and feasible, further supporting its use in complex cases such as this one [36].

## 2 Case presentation

A 30-year-old woman in her second pregnancy experienced her first episode of hypoglycemia at 13 weeks of gestation. She had been facing recurrent symptoms of palpitations and perspiration, which subsided after she consumed snacks. These symptoms were new to her as she had not experienced them before the current pregnancy and had never monitored her blood glucose levels.

In a concerning turn of events, she suffered an episode of syncope, resulting in head trauma, and was urgently taken to a hospital. Notably, she displayed neither seizures nor muscle weakness during this episode. Upon examination, her capillary blood glucose was found to be immeasurably low, and her plasma glucose level was critically low at 1.4 mmol/L. She regained consciousness following an intravenous glucose infusion, leading to a clinical diagnosis of symptomatic hypoglycemia.

Further review of her medical history revealed that she had undergone a spontaneous miscarriage at 5 weeks during her previous pregnancy. In the wake of her recent health scare, she was advised to include a sweetened beverage in her daily diet and was referred to a secondary care hospital for a thorough investigation into the underlying cause of her hypoglycemia.

### 2.1 Diagnostic assessments

At 15 weeks of gestation, the patient underwent testing at the secondary care hospital she had been referred to. She presented with a plasma glucose level of 2.9 mmol/L, an insulin level of 35.14 pmol/L, a C-peptide level of 0.26 nmol/L, and a cortisol level of 493.8 nmol/L, with the duration of fasting unknown. The preliminary diagnosis was insulinoma during pregnancy, established upon the confirmation of hypoglycemia accompanied by hyperinsulinemia.

The patient was referred to Siriraj Hospital, a tertiary care facility, for endocrinological and surgical management. At 17 weeks of gestation, her medical history underwent a comprehensive review, and a full physical examination was conducted. She had no family history of diabetes or type 1 multiple endocrine neoplasia, and the physical assessment identified no significant abnormalities. Initial laboratory findings indicated normal renal and liver functions, as well as standard total calcium and phosphorus levels. Her HbA1c was recorded at 4.5 %. Between her hypoglycemic episodes, sulfonylurea level screenings were consistently negative. Following a 6-h fast, she had another hypoglycemic event, registering a plasma glucose level of 1.8 mmol/L, insulin at 79.17 pmol/L, C-peptide at 0.51 nmol/L, and cortisol at 656.6 nmol/L. These findings solidified the diagnosis of endogenous hyperinsulinemia during pregnancy.

**Fig. 1:**
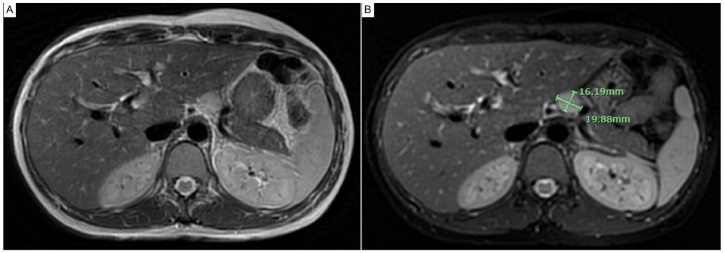
**(A)** Magnetic resonance imaging scan of pancreatic insulinoma: axial T2-weighted image alongside T2-weighted image employing fat suppression, **(B)** revealing a distinct, homogeneous mass at the superior aspect of the pancreas (approximately 2.0 × 1.6 cm).

Considering the potential risks associated with radiation exposure, iodinated contrast media, and gadolinium use during pregnancy [37], a magnetic resonance imaging scan of the pancreas was performed without gadolinium enhancement. It revealed a well-defined, homogeneous mass measuring approximately 2.0 × 1.6 cm at the superior aspect of the pancreatic body. The mass was located on the left anterolateral side of the abdominal aorta and slightly superior to the celiac axis. The anteroinferior and left lateral aspects of the mass were adjacent to the splenic artery and splenic vein, but no definite invasion was observed. The mass appeared hypointense on the T1-weighted image and hyperintense on the T2-weighted image. It also exhibited restricted diffusion on the T2-weighted image with fat suppression Fig. 1(A-B). In sum, the patient's medical history, laboratory investigations, and radiological findings confirmed the diagnosis of insulinoma during pregnancy.

### 2.2 Therapeutic interventions

Despite ongoing supportive therapy, the patient continued to experience persistent hypoglycemia, necessitating surgical intervention. At 18 weeks of gestation, transabdominal ultrasonography confirmed a single viable fetus with an estimated weight of 280 g and no detected anomalies. Intravenous glucose was administered at a steady infusion rate of 17.5 g/h to keep the capillary blood glucose levels within the optimal range of 3.9–8.3 mmol/L. At the same gestational stage, the patient underwent a robotic-assisted laparoscopic enucleation, which was enhanced with intraoperative ultrasound.

### 2.3 Surgical procedure

**Fig. 2:**
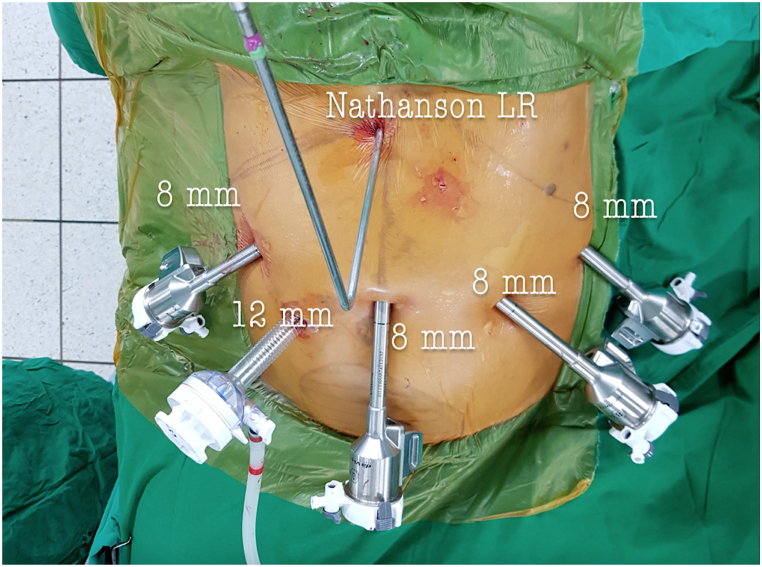
Surgical approach: configuration of port placement.

The patient was positioned supinely, after a transabdominal ultrasound confirmed fundal height, and carbon dioxide pneumoperitoneum was established using a Veress needle to maintain pressure 8–10 mmHg. A 12-mm Optiview trocar (Ethicon, Norderstedt, Germany) was positioned within the abdominal cavity, followed by the placement of additional 8-mm robotic trocars under direct camera visualization Fig. 2.

**Fig. 3:**
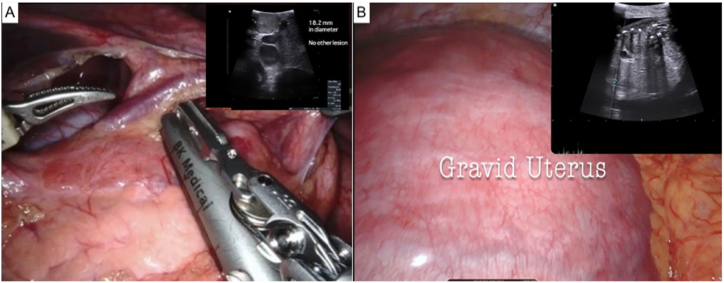
**(A)** Intraoperative ultrasound: a 1.8-cm-diameter homogeneous hypoechoic lesion. **(B)** A single viable intrauterine pregnancy.

**Fig. 4:**
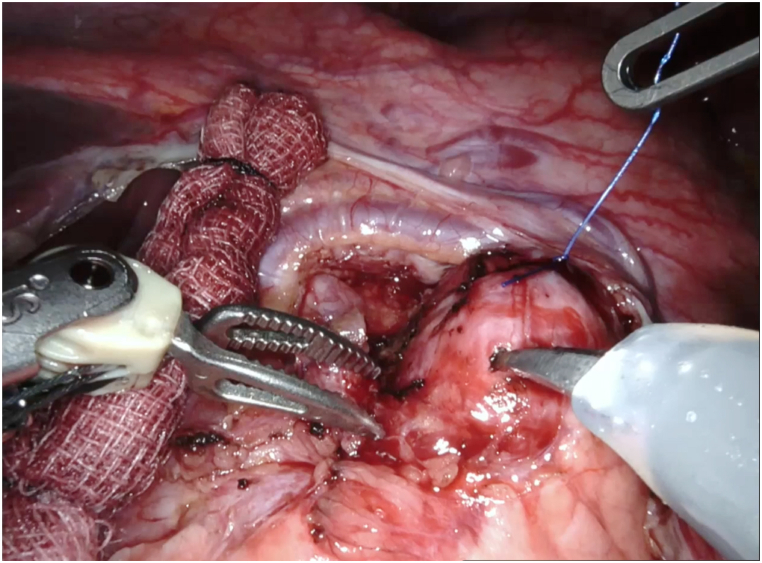
Intraoperative guidance: suture secured on tumor during enucleation process.

A da Vinci robotic cart (Intuitive Surgical, Sunnyvale, CA, USA) was positioned at the patient's right side for optimal access to the pancreatic region. Dissection of the gastrocolic ligament was performed, and an intraoperative ultrasound identified a homogenous, hypoechoic lesion measuring 1.8 cm in diameter at the pancreas as well as a viable single uterine pregnancy Fig. 3(A-B). The lesion was meticulously excised from the pancreatic tissue utilizing monopolar curved scissors and Maryland bipolar forceps, avoiding injury to the pancreatic parenchyma. A Prolene (Ethicon, Somerville, NJ, USA) 3–0 suture served as a temporary hanging aid during the excision, providing necessary traction until complete lesion removal (Fig. 4). A Jackson–Pratt drain was strategically placed within the lesser sac. Following the procedure, an intraoperative ultrasound was performed to establish the survival of a single fetus. The procedure proceeded smoothly, with a console time of 95 minutes and an estimated blood loss of 10 mL. The patient's recovery was swift, enabling her to be discharged on the fourth day following surgery.

**Fig. 5:**
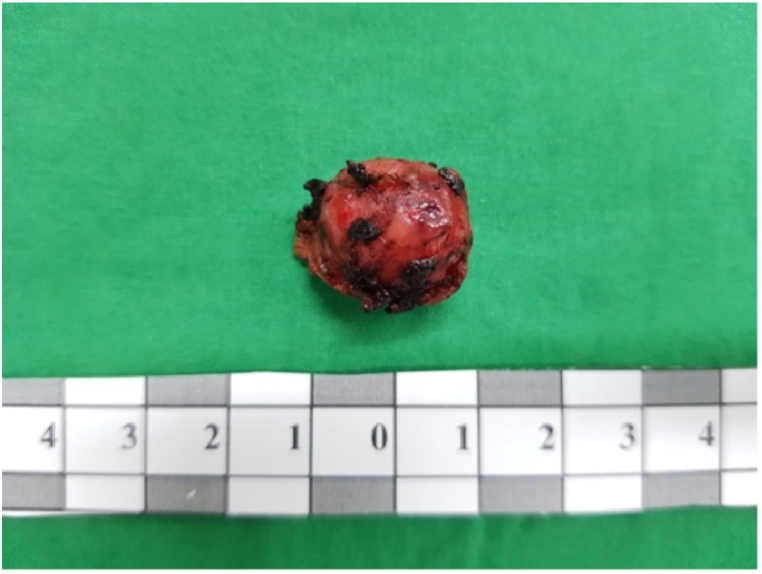
Postenucleation surgical specimen: isolated round, yellowish tumor tissue (diameter: 1.8 cm).

The excised specimen consisted of a distinct, round, yellowish mass, 1.8 cm in diameter, located at the pancreatic tail (Fig. 5). Following tumor removal, there was a notable rise in the patient's plasma glucose levels, coinciding with a reduction in both insulin and C-peptide levels (Table 1).

**Table 1:** Intraoperative laboratory investigations for insulinoma removal.

	Capillary blood glucose (mmol/L)	Plasma glucose (mmol/L)	Insulin level (pmol/L)	C-peptide level(nmol/L)	Glucose infusion rate (gm/hour)
At time of tumor manipulation	4.9				17.5
At time of tumor removal	11.9	13.2	499.31	2.02	17.5
15 minutes post-tumor removal	12.2	15.2	169.44	1.27	17.5
30 minutes post-tumor removal	14.4	15.8	172.22	1.12	12.5
45 minutes post-tumor removal	11.8				6.25

**Fig. 6:**
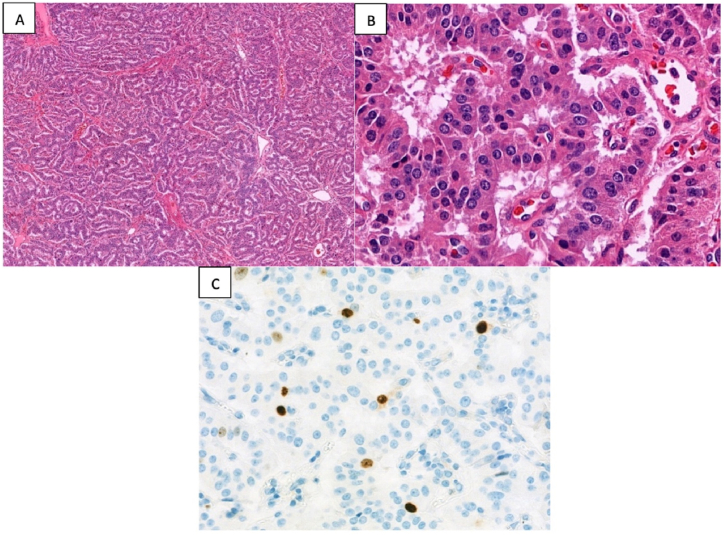
**(A)** Histological overview: low-magnification image depicting tumor cells in a ribbon/cord-like configuration amidst dispersed blood vessels. (x40, hematoxylin & eosin stained). **(B)** Cellular detail: high-magnification image showcasing eosinophilic granular cytoplasm within tumor cells. uniform nuclei — centrally positioned, ranging from round to oval — exhibit 'salt and pepper' chromatin and subtle nucleoli. (x400, hematoxylin & eosin stained). **(C)** Proliferative index: low Ki-67 labeling index (<3 %) indicating limited cellular proliferation. (x40, immunohistochemically stained).

Postoperatively, transabdominal ultrasonography was conducted at 18 weeks of gestation to assess fetal well-being. The findings indicated no anomalies, leading to the cessation of intravenous glucose. Thirteen hours after the cessation of intravenous glucose, morning blood samples revealed a plasma glucose level of 4.4 mmol/L, insulin levels of 59.58 pmol/L, and C-peptide levels of 0.42 nmol/L. The final pathological assessment disclosed a well-differentiated pancreatic neuroendocrine tumor, characteristic of an insulinoma, with dimensions of 2 x 1.5 × 1.5 cm. The tumor exhibited a nonprogressive mitotic rate, with 0 mitoses/2 mm^2^ and a Ki-67 index of less than 3 % Fig. 6(A–C).

## 3 Outcomes and follow-up

At her 2-week postoperative follow-up, the patient reported that she had not experienced any hypoglycemic symptoms since the surgery. A 12-h fasting test confirmed stable plasma glucose levels at 4.4 mmol/L, insulin at 32.71 pmol/L, and C-peptide at 0.3 nmol/L. She continued her pregnancy without encountering any episodes of hypoglycemia. She gained a healthy amount of weight, specifically 9 kg, during her pregnancy and successfully underwent a cesarean section at 38 weeks, giving birth to a healthy 3300 g infant.

Continued stability was observed at her 1-month postpartum follow-up. After 12 hours of fasting, measurements showed a plasma glucose level of 4.9 mmol/L, HbA1c at 5 %, insulin at 21.39 pmol/L, and C-peptide at 0.37 nmol/L. Further testing after 9 hours of fasting at her 4 months postpartum follow-up showed consistent plasma glucose at 4.9 mmol/L, a slightly elevated HbA1c at 5.6 %, insulin at 38.54 pmol/L, and C-peptide at 0.42 nmol/L, with no incidents of hypoglycemia reported (Table 2).

**Table 2:** Comparative laboratory analyses at initial diagnosis and during subsequent follow-up.

	Initial diagnosis	Before surgery	2 days post-surgery	2 weeks post-surgery	2 months post-surgery	1 month post-delivery	4 months post-delivery	1 year 4 months post-delivery	2 years 4 months post-delivery	3 years 4 months post-delivery
Gestational age (weeks)	15	17	18	20	29	–	–	–	–	–
Fasting time (hours)	14	6	13	12	12	12	9			
Plasma glucose (mmol/L)	2.9	1.8	4.4	4.4	4.4	4.9	4.9	4.9	4.7	5.1
Insulin level (pmol/L)	35.14	79.17	59.58	32.71	59.51	21.39	38.54	32.64	22.64	37.08
C-peptide level (nmol/L)	0.26	0.51	0.42	0.3	0.5	0.37	0.42	0.4	0.32	0.49
Cortisol level (nmol/L)	493.8	656.6	–	–	–	–	–	–	–	190.34
HbA1C (%)	–	4.5	–	4.5	4.9	5.0	5.6	5.4	5.4	5.6
Sulfonylurea levels^a^	–	negative	–	–	–	–	–	–	–	–

a Sulfonylurea profiles: levels of glipizide, gliclazide, and glimepiride.

## 4 Discussion

Insulinomas are rare, occurring in 1–4 individuals per million annually and accounting for 1%–2% of all pancreatic tumors [[38], [39], [40]]. There have been fewer than 40 reported cases of insulinoma during pregnancy worldwide [27,[29], [30], [31], [32]], with this report representing the first documented case in Thailand. Our patient, a 30-year-old woman, developed hypoglycemic episodes during the first trimester of her second pregnancy.

Early pregnancy poses a challenge for insulinoma diagnosis as the signs of hypoglycemia are often nonspecific. Prior animal research indicates that estrogen and progesterone levels rise during early pregnancy, enhancing beta-cell function [41]. However, the later stages of pregnancy see an upsurge in diabetogenic hormones, including human placental lactogen, cortisol, tumor necrosis factor-alpha, leptin, and placental-derived growth hormone. This surge potentially heightens insulin resistance [42], leading to increased blood glucose levels. In our reported case, the loss of the hyperglycemic state during early pregnancy led to a hypoglycemic episode.

In our literature review (Table 3), we found that the mean age at insulinoma diagnosis during pregnancy was 29.1 years (ranging from 19 to 41 years) [[8], [9], [10], [11], [12], [13], [14], [15], [16], [17], [18], [19], [20], [21], [22], [23], [24], [25], [26],[28], [29], [30], [31], [32]]. Our patient was diagnosed at 30, which falls within this range. Nearly half of the reviewed patients first experienced symptoms during the first trimester [[8], [9], [10], [11], [12],^14^,^16^,^18^,^20^,^22^,^24^] (42.3 %, 11/26 cases), while symptoms appeared in the postpartum period for one-third [21,23,24,26,29,30,32,43] (30.8 %, 8/26 cases). The remaining quarter manifested symptoms in the second or third trimesters [13,15,17,19,25,28,31] (26.9 %, 7/26 cases).

**Table 3:** Comprehensive review of clinical presentations, laboratory diagnostics, radiological insights, and therapeutic interventions for insulinoma during pregnancy.

Patient	Year	Age (years)	Symptom onset	Blood sugar (mmol/L)	Insulin (pmol/L)	C-peptide (nmol/L)	Pro-insulin	Radiological findings	Management
1 [8]	1977	21	Gestation: 1st trimester	2	1861.1	N/A	N/A	**Splenic angio:** 2 cm tumor in pancreatic body. During pregnancy.	Laparotomy. Gestation: week 12.
2 [9]	1983	33	Gestation: week 7	1.5	208.3–277.8	N/A	0.2 pmol/ml	**US, CT:** 2 cm tumor in pancreatic head. Both during pregnancy.	Laparotomy. Gestation: week 17.
3 [10]	1984	19	Gestation: 1st trimester	1.7	399.3	0.33	N/A	**CT, angio:** 1.8 × 2.6 cm^2^ tumor in pancreatic tail. Both during pregnancy.	Laparotomy post-pregnancy termination. 1st trimester.
4 [11]	1985	24	Gestation: week 10	1.2	201.4	1.19	0.1 μ U/ml	**CT:** no evidence of tumor. **Angio:** tumor in pancreatic body. Both post-delivery.	Laparotomy. Post-delivery.
5 [12]	1986	24	Gestation: week 6	0.6	1006.9	0.77	N/A	**US, CT, angio:** no evidence of tumor. All post-delivery.	Laparotomy. Post-delivery
6 [13]	1988	37	Gestation: week 35	1.1	631.9	N/A	N/A	**US:** no evidence of tumor. During pregnancy.	Liver examination during caesarean section showed multiple nodules of malignant insulinoma (died post-surgery day 10 due to progressive hepatic failure).
7 [14]	1988	41	Gestation: 1st trimester	1.9	437.5	N/A	N/A	**CT, angio:** no evidence of tumor. Both post-delivery.	Laparotomy. Post-delivery.
8 [15]	1990	26	Gestation: week 16	1	104.2	2.28	N/A	**CT**, **angio:** 2-cm tumor in pancreatic tail. Both post-delivery.	Laparotomy. Post-delivery.
9 [16]	1991	25	Gestation: 1st trimester	1.9	368.1	0.02	N/A	**US:** no evidence of tumor. During pregnancy.	Laparotomy. 1st trimester.
10 [17]	1992	30	Gestation: week 16	0.7	184	N/A	N/A	–	Autopsy (died 2 weeks post-delivery due to severe sepsis).
11 [18]	1992	24	Gestation: 1st trimester	1.3	54	N/A	N/A	**US:** no evidence of tumor. **MRI:** tumor in pancreatic tail. Both during pregnancy.	Exploratory laparotomy with resection of distal pancreas (2nd trimester). A 2nd laparotomy with tumor resection post-delivery.
12 [19]	1994	26	Gestation: week 16	1.7	493.1	3	N/A	**CT:** 1-cm tumor in pancreatic tail. Post-delivery.	Laparotomy. Post-delivery.
13 [20]	1994	25	Gestation: week 6	1.9	187.5	N/A	N/A	**US**, **EUS:** no evidence of tumor. Both during pregnancy.	Laparotomy. Gestation: week 17.
14 [21]	2001	36	Postpartum: day 1	1.2	N/A	N/A	N/A	**CT:** 1.5-cm tumor in pancreatic head. Post-delivery.	Laparotomy. Post-delivery.
15 [22]	2002	26	Gestation: week 6	1.3	909.7	0.03	N/A	**MRI**, **CT:** tumor in pancreatic tail. Both post-delivery.	Laparotomy. Post-delivery.
16 [23]	2002	35	Postpartum: week 3	2.1	104.2	2	N/A	**US**, **CT**, **intraarterial calcium stimulation:** no evidence of tumor. All post-delivery.	Laparotomy. Post-delivery.
17 [24]	2008	35	Postpartum: month 3	1.5	178.5	0.89	N/A	**CT**, **angiography:** no evidence of tumor. Both post-delivery.	Laparotomy. Post-delivery.
18 [24]	2008	35	Postpartum: day 26	1.9	47.2	0.29	N/A	**CT**, **EUS**, **intraarterial calcium stimulation:** no evidence of tumor. All post-delivery.	Laparoscopy. Post-delivery.
19 [24]	2008	22	Gestation: month 2	2.3	477.1	N/A	N/A	**MRI:** 2.5-cm cystic tumor in pancreatic tail. **EUS:** 1.2 × 2 cm^2^ cystic tumor and 1.7 × 1.5 cm^2^ solid hypoechoic lesion. Both post-delivery.	Laparoscopy. Post-delivery.
20 [25]	2008	29	Gestation: week 35	0.9	51	0.76	45 pmol/l	**US**, **CT**, **MRI**, **somatostatin receptor scintigraphy:** no evidence of tumor. **Intraoperative US:** 5-mm tumor in pancreatic head. All post-delivery.	Laparotomy. Post-delivery.
21 [26]	2008	26	Postpartum: day 3	1.3	38.9	0.35	16 pmol/l	**MRI:** 1.2-cm tumor in pancreatic head. Post-delivery.	Laparotomy. Post-delivery.
22 [28]	2012	29	Gestation: week 17	1.6	N/A	N/A	N/A	**US:** innumerable hypoechoic liver lesions. **MRI:** innumerable T2-hyperintense lesions throughout liver. Both during pregnancy.	Therapy with everolimus. Post-delivery.
23 [29]	2012	21	Postpartum: day 8	2.1	54	N/A	N/A	**CT:** 2-cm tumor in pancreatic head. Post-delivery.	Laparoscopic enucleation of tumor. Post-delivery.
24 [30]	2015	38	Postpartum: week 3	1.5	67.4	0.52	N/A	**US:** no evidence of tumor. **EUS:** hypoechoic lesion (8 × 9 mm^2^) in pancreatic head. Both post-delivery.	Laparotomy. Post-delivery
25 [31]	2017	36	Gestation: week 17	2.7	29.5	0.54	7.49 μ U/ml	**EUS:** 1.8 × 2.2 cm^2^ tumor in pancreatic tail. **MRI:** 2-cm hyperintense solid lesion in pancreatic tail. Both during pregnancy.	Laparotomy in 2nd trimester. Gestation: week 21.
26 [32]	2020	34	Postpartum: day 2	2	300	1.62	N/A	**US:** no evidence of tumor. **MRI:** 10 × 8 mm^2^ lesion in pancreatic head. **EUS:** 14.3 mm lesion in pancreatic head. All post-delivery.	Laparotomy. Post-delivery.
27 Our case	2020	30	Gestation: week 13	1.4	79.2	0.51	N/A	**MRI:** 2.0 × 1.6 cm^2^ tumor in pancreatic body. During pregnancy.	Robotic-assisted pancreatic enucleation. Gestation: week 18.

Angio, angiography; CT, computed tomography; EUS, endoscopic ultrasound; MRI, magnetic resonance imaging; N/A, not available; US, ultrasound.

Blood glucose levels at diagnosis varied, ranging from 0.6 to 2.7 mmol/L, with insulin levels between 38.89 and 1861.11 pmol/L and C-peptide levels from 0.02 to 3 nmol/L [[8], [9], [10], [11], [12], [13], [14], [15], [16], [17], [18], [19], [20], [21], [22], [23], [24], [25], [26],[28], [29], [30], [31], [32]]. Our patient presented with plasma glucose levels of 2.9 mmol/L, insulin levels of 35.14 pmol/L, and C-peptide levels of 0.26 nmol/L.

For insulinoma localization during pregnancy, endoscopic ultrasound and magnetic resonance imaging are the most commonly used methods. They are preferred due to their low radiation exposure and the avoidance of iodinated contrast media, which can pose potential risks during pregnancy [[8], [9], [10], [11], [12], [13], [14], [15], [16], [17], [18], [19], [20], [21], [22], [23], [24], [25], [26],[28], [29], [30], [31], [32]].

Treatment for insulinoma encompasses supportive measures and specific therapies. Patients are advised to consume frequent, small meals rich in carbohydrates [44] and to recognize and appropriately react to signs of hypoglycemia. Available pharmacological interventions include diazoxide, somatostatin analogs, and corticosteroids, all aimed at managing and preventing hypoglycemia [45]. Nonetheless, their use during pregnancy requires caution.

Regarding diazoxide, there is a lack of controlled data on its use in human pregnancy. Animal studies have shown potential fetal pancreatic beta-cell degeneration [46]. Octreotide acetate, another treatment option, lacks established safety data in pregnancy due to the absence of substantial, well-controlled studies in pregnant women. However, there is one case report linking octreotide usage during pregnancy to fetal growth restriction in a patient with symptomatic familial hyperinsulinemic hypoglycemia [47]. Corticosteroid usage in the first trimester may increase the risk of cleft lip with or without cleft palate. However, evidence regarding systemic corticosteroid use in pregnancy and its impact on preterm delivery, low birth weight, or preeclampsia is limited [48].

Surgery remains the optimal choice for insulinoma, providing symptomatic relief and a potential long-term solution [44]. Various surgical methods, including open, laparoscopic, and robotic techniques, are employed. Both open and laparoscopic procedures are underresearched because cases are rare [49]. For benign, small, and solitary pancreatic tumors [50], laparoscopic enucleation is recommended since it offers reduced hospitalization and quicker recovery than open surgery [51]. Robotic enucleation has proven advantageous in terms of shorter surgery duration, minimal blood loss, and similar rates of major postoperative complications compared to other methods [52,53]. Given the enhanced ergonomics robotic systems offer surgeons, coupled with dependable 3D imaging, superior maneuverability in tight spaces, and the ability for precise tissue manipulation, we foresee numerous advantages for pregnant patients. In the context of pregnant individuals with insulinoma, robotic enucleation could prove beneficial for both the mother and the child. To the best of our knowledge, no research has shown this surgical procedure in pregnancy.

The decision to use robotic surgery was influenced by several key factors. Robotic surgery provides enhanced three-dimensional visualization and improved dexterity, offering up to seven degrees of freedom. These features are particularly beneficial for complex procedures like pancreatic enucleation. Additionally, robotic systems help to standardize and smooth surgical motions, eliminating tremors and improving precision. According to the International consensus guidelines on robotic pancreatic surgery in 2023, robotic pancreatic enucleation is effective for superficial benign tumors and is associated with fewer conversions to open surgery compared to laparoscopic methods [54]. It also offers less trauma, quicker wound recovery, reduced intraoperative blood loss, shorter hospital stays, shorter operative times, and fewer overall complications. Given these advantages and the complexity of the procedure, robotic surgery was deemed the most suitable option.

Although this marked our first case of robotic pancreatic enucleation in a pregnant patient, literature reviews suggest that robotic surgery is safe and feasible during pregnancy [36]. Moreover, as a medical school with specialized departments, including anesthesiology, the team involved had significant experience in administering anesthesia to pregnant patients, ensuring the safety and well-being of both the mother and the fetus. During surgical intervention in pregnancy, it is imperative to prioritize fetal health. In this context, our patient underwent continuous maternal and fetal health monitoring by an obstetrician throughout the surgical process. No anomalies were detected.

A retrospective study examined 77 pregnant patients who underwent nonobstetric abdominal surgery from 1989 to 1996. The findings showed no significant association between surgery performed during the first or second trimesters and increased rates of preterm labor, fetal loss, or teratogenicity. However, surgery in the third trimester correlated with a higher incidence of preterm labor [55]. Our patient, diagnosed in the second trimester, underwent surgical treatment at 18 weeks of gestation.

Among insulinoma patients during pregnancy, prior research found that the predominant treatment was laparotomy and tumor excision (accounting for 21/26 cases or 80.8 %), followed by laparoscopy in 3/26 cases (11.5 %). Surgical interventions were distributed as follows: first trimester (3/26 cases, 11.5 %), second trimester (4/26 cases, 15.4 %), postpartum period (17/26 cases, 65.4 %), and cesarean section (1/26 cases, 3.8 %) [[8], [9], [10], [11], [12], [13], [14], [15], [16], [17], [18], [19], [20], [21], [22], [23], [24], [25], [26],[28], [29], [30], [31], [32]]. A case review [56] indicated successful outcomes with laparotomy conducted in three instances in the first trimester, three in the second trimester, and two during or immediately following cesarean delivery. Each scenario resulted in favorable maternal and neonatal outcomes. Despite the fact that a minimally invasive method such as laparoscopy is acceptable and is safe and successful, no laparoscopic treatment for insulinoma during pregnancy has been recorded. While nonobstetrical robotic-assisted surgery in pregnant women is uncommon, no unfavorable maternal-fetal outcomes have been recorded in the instances analyzed [36]. Our report documents the first instance in Thailand of the successful management of insulinoma during pregnancy through the use of robotic-assisted pancreatic enucleation.

## 5 Conclusion

For selected pregnant patients with insulinoma, a minimally invasive approach as robotic surgery is safe and feasible. Such innovative techniques have the potential to benefit both mothers and fetuses. Further study and follow-up over a long length of time are required to establish the advantages.

## Ethics statement

The authors confirmed that the patient provided informed consent for the publication of her anonymized case details and images.

## Data availability statement

Data included in the article, supplementary material, or referenced in the article for this manuscript.

## CRediT authorship contribution statement

**Voraboot Taweerutchana:** Writing – review & editing, Writing – original draft, Visualization, Validation, Supervision, Methodology, Investigation, Formal analysis, Data curation, Conceptualization. **Sawaraj Choksakunwong:** Writing – review & editing, Visualization, Validation, Supervision, Resources, Methodology, Investigation, Formal analysis. **Amornrat Lerwattrakarn:** Writing – review & editing, Validation, Supervision, Resources, Methodology, Investigation, Formal analysis. **Wipapat Vicki Chalermwai:** Writing – review & editing, Visualization, Validation, Supervision, Resources, Methodology, Investigation, Formal analysis. **Thita Intralawan:** Writing – review & editing, Visualization, Validation, Supervision, Software, Resources, Methodology, Investigation, Formal analysis. **Raweewan Lertwattanarak:** Writing – review & editing, Validation, Supervision, Software, Resources, Project administration, Funding acquisition, Formal analysis, Conceptualization.

## Declaration of competing interest

The authors declare that they have no known competing financial interests or personal relationships that could have appeared to influence the work reported in this paper.
